# Changes in Left Ventricular Ejection Fraction and Clinical Trajectories of Transthyretin Cardiac Amyloidosis with Systolic Dysfunction

**DOI:** 10.3390/jcm12237250

**Published:** 2023-11-23

**Authors:** Joshua Saef, Trejeeve Martyn, Anusha Ray Dey, Rola Khedraki, Lauren Ives, Patrick Collier, Wael A. Jaber, Jerry D. Estep, Mazen Hanna, Wai Hong Wilson Tang

**Affiliations:** 1 Department of Cardiovascular Medicine, Heart, Vascular and Thoracic Institute, Cleveland Clinic, Cleveland, OH 44195, USA; jsaef@mhs.net (J.S.); martynm2@ccf.org (T.M.); hannam@ccf.org (M.H.); 2 Joe DiMaggio Children’s Hospital and the Memorial Healthcare Total Heart Center, Hollywood, FL 33021, USA; 3 Department of Medical Science, Brown University, Providence, RI 02912, USA; 4 Division of Cardiology, Scripps Clinic and Research Foundation, La Jolla, CA 92037, USA; 5 Department of Cardiovascular Medicine, Heart, Vascular and Thoracic Institute, Cleveland Clinic Florida, Weston, FL 33331, USA

**Keywords:** Transthyretin amyloid cardiomyopathy, heart failure with reduced ejection fraction, reverse remodeling, prognosis

## Abstract

Background: Transthyretin cardiac amyloidosis (ATTR-CM) is classically thought of as a progressive disease with preserved systolic function. The longitudinal clinical trajectories of ATTR-CM with impaired left ventricular ejection fraction (LVEF) remain unclear. Methods: This is a single-center retrospective cohort study of consecutive patients with ATTR-CM who underwent two or more echocardiograms with baseline LVEF < 50%. Patients were stratified according to the presence of ≥5% change in LVEF. A Cox proportional hazard model examined hazard of a composite outcome of death, transplant, or LVAD insertion over the two years following diagnosis. Results: In our study cohort of 179 patients, 62 patients (34.6%) experienced an increase in LVEF while 33 (18.4%) experienced a decrease in LVEF. After adjusting for covariates, patients with a decrease in EF experienced increased hazard of death (HR 2.15, 95% CI 1.05–4.40, *p* = 0.038) compared to those with stable or an increase in LVEF. Changes in LVEF corresponded with significant differences in NT proBNP trajectories, but initial biomarker levels or clinical staging were not predictive of LVEF trajectory. Conclusions: in ATTR-CM patients with impaired LVEF, over a third demonstrated improved LVEF over time, while those with a decrease in LVEF had worse long-term outcomes.

## 1. Introduction

Cardiac amyloidosis (CA) is characterized by the extracellular deposition of amyloid fibrils in the heart, with the distinctive histological property of green birefringence when viewed under cross-polarized light microscopy after staining with Congo red. There are two main types of CA, namely transthyretin (TTR) cardiomyopathy (ATTR-CM) and light chain (AL) cardiomyopathy (AL-CM) [1]. In ATTR-CM, the native tetrameric form of TTR, produced mainly in the liver, pathologically dissociates to form amyloid fibrils that deposit in the myocardium, nerves, and soft tissues, and peripheral and/or autonomic neuropathy. The most common variant in the United States is the valine to isoleucine substitution at amino acid 122 (V122I or p.V142I), of which 3% to 4% of African Americans are heterozygote carriers, putting them at risk for the phenotypic expression of late-onset ATTR-CM. The Median survival after diagnosis in untreated patients is poor: 2.5 years for ATTRv-CM caused by the V122I variant and 3.6 years for ATTRwt-CM [2].

Transthyretin amyloid cardiomyopathy (ATTR-CM) was previously considered a rare disease, but now has a growing recognition as a potential treatable cause of heart failure (HF), especially in older adults [3]. Although described predominantly as a disease of heart failure with preserved ejection fraction (HFpEF), ATTR-CM can frequently present as heart failure with systolic dysfunction, including mildly reduced (HFmrEF, left ventricular ejection fraction [LVEF] 41–49%) and reduced ejection fraction (HFrEF, LVEF ≤ 40) [4]. A recent analysis from our institutional registry reported that 45% of those newly diagnosed with ATTR-CM had an LVEF less than 50%, with 28% presenting with HFrEF [2]. Therefore, the HFmrEF/HFrEF phenotype in ATTR-CM is quite common, yet the clinical trajectory of those with abnormal LV function is poorly characterized. Conventional wisdom regarding the natural history of ATTR-CM is that the onset of diastolic dysfunction and HF symptoms precedes systolic dysfunction; hence, evidence of systolic dysfunction has been postulated to reflect progressive advanced disease that may become irreversible [5].

The latest American College of Cardiology/American Heart Association clinical guidelines in the management of heart failure have provided new recommendations regarding the diagnosis and treatment of ATTR-CM. Specifically, patients with ATTR-CM presenting with HFmrEF/HFrEF should cautiously follow general recommendations of initiating guideline-directed medical therapy, despite little prospective evidence to guide management [6]. Given the progressive infiltrative nature of ATTR-CM, there is uncertainty around whether LV recovery is possible following medical management/optimization and if such positive remodeling is clinically meaningful [7,8]. Herein, we sought to investigate the prevalence, clinical trajectories, and prognostic significance of LVEF changes in patients with ATTR-CM with impaired LVEF at the time of diagnosis. 

## 2. Materials and Methods

### 2.1. Study Population

We included consecutive ATTR-CM patients with HFrEF (defined as LVEF ≤ 40% using the Simpson’s Biplane Method) or mildly reduced ejection fraction (HFmrEF, defined as LVEF 41–49% using the Simpson’s Biplane Method) seen at a tertiary care hospital system between 1 January 2008 and 31 May 2021 with available serial echocardiographic evaluations performed. The protocol was approved by our Institutional Review Board as a retrospective study that waived informed consent. 

### 2.2. Data Synthesis

ATTR-CM patients over age 18 years with either HFrEF or HFmrEF were considered under the umbrella of HF with abnormal LVEF (HFmrEF/HFrEF). Diagnosis of ATTR-CM was established via endomyocardial biopsy or with 99mTc-PYP scanning with single-photon positron emission computerized tomography (SPECT)/computed tomography (CT). A 99mTc-PYP was considered positive based on a heart to contralateral ratio of 1.3 or greater at 3 h and myocardial uptake grade 2 or 3. Patients were confirmed to have ATTR-CM through the appropriate exclusion of light chain (AL) amyloidosis based on standard guideline-based criteria. Demographic, clinical characteristic, imaging, and laboratory data were obtained at the initial clinical encounter. Baseline LVEF was adjudicated from echocardiogram performed closest to the time of diagnosis within 90 days of the initial clinical encounter [9]. Follow-up LVEF was determined via echocardiographic assessment at least 3 months and at most 12 months after baseline exam. We recognized that the standard deviation and margin of error in estimating LVEF by echocardiogram reached ±5%. Therefore, patients were arbitrarily stratified into those who experienced worsened LVEF (reduction in LVEF of ≥5%), stable LVEF (defined as change in LVEF of <5%), or improved LVEF (defined as an increase in LVEF of ≥5%). The primary outcomes were death, heart transplant, or left ventricular assist device, which were adjudicated by manual chart review. Demographic, clinical characteristic, imaging, and laboratory data were abstracted from the electronic health record (Epic Systems, Verona, WI, USA) at the initial clinical encounter, making use of our institutional ATTR-CM clinical registry. 

### 2.3. Statistical Analysis

Data were expressed as medians (interquartile ranges (IQR)) given non-normal distributions and proportions where appropriate. Between-group comparisons of baseline comparisons were performed using the chi-squared test or Fisher Exact testing as appropriate for categorical variables and Kruskal–Wallis testing for continuous variables. Time to event was visualized using Kaplan–Meier curves, and group differences were tested with the Log-rank test. Cox proportional hazard models were then used to examine hazard of death, transplant, or left ventricular assist device (LVAD) following diagnosis with ATTR-CM. Penalized smoothing (p) splines were used to account for nonlinear relationships between covariates and hazard. Schoenfeld testing was performed to validate that the proportional hazards assumption was maintained. Survival comparisons were performed across groups and adjusted for race, NT-proBNP, cardiac troponin-T, baseline LVEF, and coronary artery disease. Statistical analyses were performed using R Studio Statistical Software version 1.2.5033 (Foundation for Statistical Computing, Vienna, Austria), and *p* value < 0.05 was considered statistically significant.

## 3. Results

In our contemporary cohort of 262 consecutive patients with ATTR-CM and systolic dysfunction (165 with HFrEF/97 with HFmrEF), 179 had sequential echocardiograms performed with median duration of 12.4 months between echocardiograms. We observed that 62 patients (34.6%) demonstrated improvement in LVEF, 84 patients (46%) had stable or unchanged LVEF, and 33 patients (18.4%) experienced worsened LVEF over the first year following their baseline assessment (Table 1). For those that demonstrated improvement in LVEF (increase 5+ %), the median LVEF change was 15% (IQR 8% to 24%), which was more prominent in those with HFrEF (median 20% (IQR 11–25%)) than HFmrEF (median 9.5% (IQR 7% to 15%)) as expected. There were no significant differences in demographics across the three groups. The baseline medication prescriptions did not show predictive value of LVEF trajectory. Interestingly, we observed that serial changes in NT-proBNP tracked with changes in LVEF. Specifically, patients with a decrease in EF experienced significantly increased in NT-proBNP levels over time. However, there were no significant differences in baseline cardio-renal biomarkers or distribution of National Amyloidosis Center ATTR-CM clinical staging across groups (Table 1). About 63% of patients in the analysis received tafamidis during their course at our institution, though the timing after diagnosis and duration of therapy prior to follow-up echocardiography were variable. The proportion of patients who received tafamidis was similar between those with a decrease in LVEF versus those with stability or an increase in LVEF (57.1% vs. 63.5%, *p* = 0.613).

**Figure 1:**
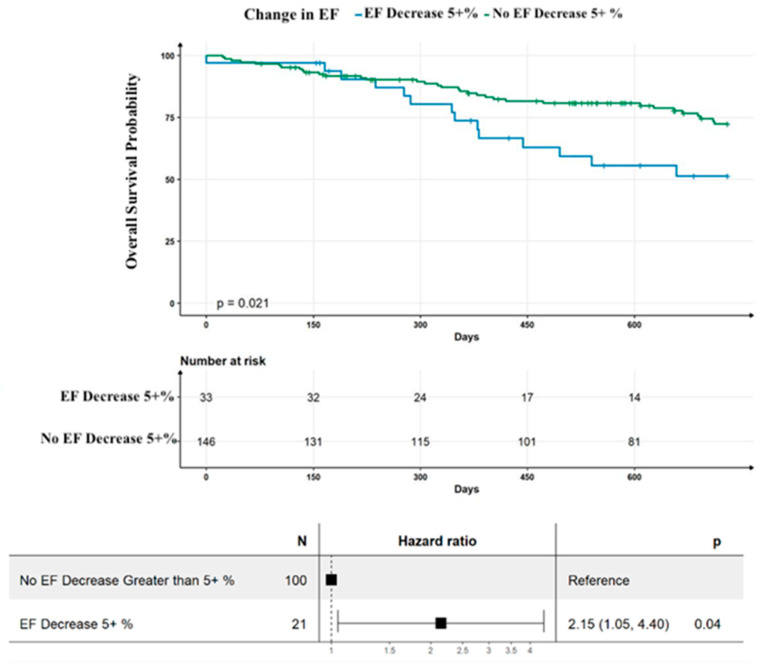
Five-year Kaplan–Meier Curves showing transplant/LVAD free survival for all 179 patients. Forest plot for Cox proportional hazard model assessing survival in those with decrease in ejection fraction (EF) versus all other patients that adjusts for adjusted for race, NT-proBNP, cardiac troponin-T, baseline LVEF, and coronary artery disease. (*n* = 121, 60 events). Black squares represent hazard ratio estimates.

The Kaplan–Meier curve comparing survival between those with a reduction in LVEF implied differences in survival from those with stable or increased in LVEF. The log rank test was consistent with a significant difference, with a *p*-value of 0.021. After adjusting for covariates, patients with a reduction in LVEF experienced worse outcomes compared to those with stable or increased in LVEF over a five-year follow-up period (HR 2.15, 95%CI 1.05–4.40, *p* = 0.04) (Figure 1). 

## 4. Discussion

In our single-center retrospective cohort in tertiary referral center, we observed that over a third of ATTR-CM patients with HFmrEF or HFrEF experienced unchanged or increased LVEF over time, and had better outcomes than those with a decrease in LVEF over time. There were no specific baseline clinical characteristics that were associated with a greater likelihood of increase in LVEF over time. Taken together, we observed that the stability or improvement of left ventricular function portends a better prognosis than those whose LVEF declines after adjusting for key clinical prognostic variables. 

Transthyretin amyloid cardiomyopathy is classically described as a form of HFpEF with adverse cardiac remodeling because of progressive amyloid fibril deposition, leading to cardiac hypertrophy and diastolic dysfunction, restrictive cardiomyopathy, and ultimately systolic dysfunction as a harbinger of advanced disease that is presumably irreversible [5]. Our center’s diverse cohort, through the broad adoption of a standardized evaluation process including advanced cardiac imaging and/or endomyocardial biopsy, has gained novel insights into a more prevalent HFrEF/HFmrEF clinical presentation of cardiac amyloidosis. We previously reported that up to 28% of patients with ATTR-CM had LVEF ≤ 40, and 45% had an LVEF < 50% at the time of presentation [4]. This was clinically relevant, as ATTR-CM may not be part of the differential diagnosis for patients presenting with HfrEF. It is interesting to note that the propensity for reverse remodeling did not track with clinical staging for ATTR-CM, suggesting that ATTR-CM with systolic dysfunction may be a heterogeneous patient cohort with a subset that represents advanced end-stage ATTR-CM, while others can be stabilized and potentially reversible over time, despite having ATTR-CM. This implies that individualized evaluation and judicious medical management, possibly with guideline-directed medical therapy, should be considered.

Although the role of guideline-directed medical therapy in ATTR-CM is still debated, the fact that some patients improve their LV function with medical management is encouraging and merits further study [7,8]. The evidence for conventional guideline-directed medical therapy in patients with ATTR-CM is mixed. Clinical evidence on traditional HF therapies with treatments such as beta-blockade, mineralocorticoid receptor antagonists (MRA), and renin-angiotensin-aldosterone inhibition (RAASi) in ATTR-CM is limited. There is a wide variation in practice and little definitive evidence to guide the management of patients with definitive ATTR-CM, particularly those with left ventricular systolic dysfunction. The presence of autonomic dysfunction, conduction disease, and the inability to augment stroke volume in response to vasodilation are particular concerns with neurohormonal blockade in these patients [7]. A 2021 publication from Cheng and colleagues retrospectively examined the association of neurohormonal blockade and adjusted ATTR-CM outcomes in a longitudinal cohort. In their analysis, there was no association between the use of beta-blockade or RAASi and mortality in patients with ATTR-CM. Additionally, they report an association between beta-blocker discontinuation and survival. In a much larger study from the National Amyloidosis Centre, a propensity score-matched analysis demonstrated that treatment with MRAs was independently associated with a reduced risk of mortality in the overall population and in a pre-specified subgroup of patients with a left ventricular ejection fraction (LVEF) >40. Furthermore, treatment with low-dose beta-blockers was independently associated with a reduced risk of mortality in a pre-specified subgroup of patients with a LVEF ≤40%. There were no convincing differences between outcomes of treatment with ACEi/ARBs [8] 

Whether this reverse remodeling is secondary to the implementation of disease-modifying therapies for systolic heart failure or adjustments in loop and thiazide diuretics leading to adequate decongestion is unclear based on these results. The study period mostly pre-dates the advent of contemporary-guideline-directed medical therapy (GDMT) medications, which includes angiotensin receptor/neprilysin inhibitor and sodium-glucose co-transporter 2 inhibitors; therefore, further investigation into the impact of contemporary GDMT on ATTR-CM is needed [9,10]. Future studies should characterize the impact of GDMT in the ATTR-CM population with regards to systolic function, cardiac biomarkers, and clinical outcomes over time.

The significant differences in natriuretic peptide trajectories and their association with prognosis and LV recovery suggests a potential role for natriuretic peptides not only with the initial staging of ATTR-CM, but also in the ongoing assessment of clinical trajectory, including the presence of positive remodeling [11]. In contrast, a lack of clinical response within the first year of diagnosis (in terms of worsening LVEF or NT-proBNP) should warrant considerations of advanced therapies if eligible. Certainly, in an elderly population with significant co-morbidities there may be a “multi-hit” hypothesis of LV systolic dysfunction, wherein co-morbid hypertension, diabetes, atrial fibrillation, and CAD could lead to systolic dysfunction that is responsive to conventional HF management in selected patients. Further phenotyping and prospective research into the optimal medical management of HF in patients with ATTR-CM is needed, particularly given the advent of effective disease-modifying treatments for the underlying disease [12,13]. 

Limitations: Our single-center retrospective cohort in a tertiary referral center is limited by the expected selection and referral biases that may impact a larger-than-expected proportion of HFrEF presentation. Patients also had varying time intervals of clinical follow-up evaluations and incomplete availability and timing of repeat echocardiograms and biomarkers. While this analysis showed prognostic associations with systolic function over time and the ability of amyloid ventricles to recover function, it did not provide further insight into which patients are likely to experience improvement or deterioration in LVEF. Whether this is reflective of the genotype, volume optimization, medical therapy use, burden of amyloid deposition, acuity of presentation, or other factors should be the subject of further study.

## 5. Conclusions

An increase in LVEF is observed in over a third of ATTR-CM patients with systolic dysfunction within the first year. Those who experienced decrease in LVEF had a two-fold increased hazard of worse long-term outcomes than those with increased or unchanged LVEF, suggesting potential insights for medical management and longitudinal risk stratification in ATTR-CM with systolic dysfunction. Future studies should prospectively address the role of contemporary GDMT in the clinical outcomes of patients living with ATTR-CM. 

## Figures and Tables

**Table 1:** Baseline characteristics stratified using ejection fraction (EF) trajectory.

	EF Decrease 5+ %	No EF Change 5+ %	EF Increase 5+ %	*p* Value
*n*	33	84	62	
Age	78.00 [71.00, 80.00]	77.00 [71.00, 81.00]	72.00 [68.00, 80.00]	0.136
Male sex	28 (84.8)	77 (91.7)	56 (90.3)	0.54
White race	18 (54.5)	49 (58.3)	45 (72.6)	0.122
Hypertension	27 (81.8)	63 (75.0)	47 (75.8)	0.726
Diabetes	9 (27.3)	21 (25.0)	12 (19.4)	0.619
Coronary artery disease	15 (46.9)	35 (41.7)	29 (46.8)	0.789
Coronary interventions	8 (53.3)	17 (48.5)	14 (51.8)	0.903
Atrial fibrillation/flutter	20 (60.6)	46 (54.8)	44 (71.0)	0.138
NYHA functional class				0.344
1	0 (0.0)	6 (8.3)	3 (5.6)	
2	11 (37.9)	36 (50.0)	28 (51.9)	
3	16 (55.2)	28 (38.9)	19 (35.2)	
4	2 (6.9)	2 (2.8)	4 (7.4)	
Body mass index (kg/m^2^)				0.936
>30	8 (24.2)	22 (26.2)	13 (21.0)	
25–30	16 (48.5)	36 (42.9)	30 (48.4)	
<25	9 (27.3)	26 (31.0)	19 (30.6)	
eGFR (ml/min/1.73 m^2^)	54.50 [40.59, 69.25]	60.09 [48.12, 74.21]	59.50 [47.25, 74.75]	0.242
Cardiac troponin T (ng/mL)	0.07 [0.04, 0.11]	0.04 [0.02, 0.10]	0.04 [0.02, 0.09]	0.141
Baseline NT-proBNP (pg/mL)	3063.00 [2287.00, 4250.00]	2665.00 [1616.25, 5506.50]	3068.50 [1763.75, 5172.00]	0.745
NT-proBNP change (pg/mL)	+2099.50 [142.50, 8772.25]	−85.00 [−699.75, 918.50]	+186.00 [−612.00, 2557.00]	0.004
ACE inhibitors	12 (42.9)	22 (32.4)	14 (24.6)	0.226
ARB	8 (28.6)	12 (17.4)	10 (17.9)	0.417
Beta Blocker	19 (65.5)	28 (40.6)	23 (41.1)	0.055
NAC Staging (%)				0.752
1	7 (38.9)	18 (43.9)	16 (43.2)	
2	6 (33.3)	17 (41.5)	16 (43.2)	
3	5 (27.8)	6 (14.6)	5 (13.5)	

Data are presented as median (interquartile range) or *n* (%). Abbreviations: eGFR, estimated glomerular filtration rate; NT-proBNP, aminoterminal pro-B-type natriuretic peptide; ACE, angiotensin-converting enzyme; ARB, angiotensin receptor blocker; NAC, National Amyloidosis Center.

## Data Availability

The data that support the findings of this study are available from the corresponding author upon reasonable request.
